# Supplementary material for: LARP7 suppresses P-TEFb activity to inhibit breast cancer progression and metastasis
Source: eLife. 2014 Jul 22;3:e02907. doi: 10.7554/eLife.02907 (PMC4126343; doi:10.7554/eLife.02907)
Supplement: Supplementary file 1. — List of qRT-PCR primers. DOI: http://dx.doi.org/10.7554/eLife.02907.013 [file elife02907s001.docx]

**Supplementary file 1A. qRT-PCR primers for analyzing RNA levels of specific genes.**

| Gene | Forward | Reverse |
| --- | --- | --- |
| 7SK | TTGATCGCCAGGGTTGATT | GGGATGGTCGTCCTCTTCG |
| LARP7 | TAATTGCCAGAGCATTGAGA | ACATACACTGTGCGTTCATC |
| E-cadherin | GCCCCATCAGGCCTCCGTTT | ACCTTGCCTTCTTTGTCTTTGTTGGA |
| DSP | GCAGGATGTACTATTCTCGGC | CCTGGATGGTGTTCTGGTTCT |
| KRT19 | AACGGCGAGCTAGAGGTGA | GGATGGTCGTGTAGTAGTGGC |
| MMP2 | GATACCCCTTTGACGGTAAGGA | CCTTCTCCCAAGGTCCATAGC |
| MMP9 | AGACCTGGGCAGATTCCAAAC | CGGCAAGTCTTCCGAGTAGT |
| N-cadherin | ACAGTGGCCACCTACAAAGG | CCGAGATGGGGTTGATAATG |
| Vimentin | GAGAACTTTGCCGTTGAAGC | GCTTCCTGTAGGTGGCAATC |
| Oct4 | CGGAGGAGTCCCAGGACATCA | TGGTCGTTTGGCTGAATACCTT |
| Sox2 | GCCGAGTGGAAACTTTTGTCG | GGCAGCGTGTACTTATCCTTCT |
| ALDH1 | CCGTGGCGTACTATGGATGC | GCAGCAGACGATCTCTTTCGAT |
| ZEB1 | AGTGGTCATGATGAAAATGGAACACCA | AGGTGTAACTGCACAGGGAGCA |
| ZEB2 | GACAGATCAGCACCAAATGC | GCTGATGTGCGAACTGTAGG |
| Twist1 | GAAAAGCGAGACAGGCCCGTG | GATTGGCACGACCTCTTGAG |
| Twist2 | GGCGCGCCAGGAGGAGATTCT | TGCTCACTCCCGCCAACGTT |
| FOXC2 | GCCTAAGGACCTGGTGAAGC | TTGACGAAGCACTCGTTGAG |
| Snail | TGCGCGAATCGGCGACCC | CTAGAGAACCGCTTCCCGCAG |
| Slug | CTGGGCGCCCTGAACATGCAT | GGCTTCTCCCCCGTGTGAGTTCTA |
| Sox10 | CCTCACAGATCGCCTACACC | CATATAGGAGAAGGCCGAGTAGA |
| AF9 | AGTGCCTTCAAAGAACCTTCC | GTCCACTGGTGATGGTGAGTA |
| ELL2 | CATCACCGTACTGCATGTGAA | ACTGGATTGAAGGTCGAAAAGG |
| AFF4 | ACCTCCAACCCTAACAAGCC | TTTCCATAGTGTCTTGAGCACC |
| β-actin | GATCATTGCTCCTCCTGAGC | ACTCCTGCTTGCTGATCCAC |

**Supplementary file 1B. qRT-PCR primers for ChIP analyses.**

| Name | Forward | Reverse |
| --- | --- | --- |
| FOXC2: TSS | CCCTCCCGCTCCCCTCCTCT | CTGCTTCCGAGACGGCTCGC |
| FOXC2: interior | AGAACAGCATCCGCCACAACC | CCGGGTCCAGGGTCCAGTAA |
| FOXC2: 3’-UTR | CTCGGGGAGTCCCAGGTGAG | TTTCGTGCAGTCGTAGGAGTAGGG |
| Twist1: TSS | CCCCGCTCTTCTCCTCTGCC | GTTGCTCAGGCTGTCGTCGG |
| Twist1: interior | CGCTCAGCCTTCCCACCTCA | CGGTCCTTACCCGTGACCCT |
| Twist1: 3’-UTR | TTGGTGGTAAATACCTGCTTG | TGCCTGTCAGTAGCTGCTTT |
| ZEB2: TSS | TAGATCGAGCCTGCGTGCTG | CCTGGGATTGGCTTGTTTGC |
| ZEB2: interior | GTTTGGTAGGTCCGTTTTGG | CACGATTTTGTTTGGGGTAAT |
| ZEB2: 3’-UTR | TAAACATGGAGATAATTGGCAACA | TTCAACCCTGAAACAGAGGC |
| Slug: TSS | CCAGAGGGAGGAGCTGAAAT | TTACGAACTGAGCCCGTTTT |
| Slug: interior | CAGGAGCATACAGCCCCATCA | GGGACTCACTCGCCCCAAAGA |
| Slug: 3’-UTR | CAGTATTGCTTTGTAATAGAGTC | CATGTAATGGAGTAAACTTGG |
| Snail: TSS | AGTACTTAAGGGAGTTGGCGG | TTCCTGACGAGGAAAGAGCG |
| Snail: interior | GCTTTTTGGTTGGAGCAGGG | AGCTGGGTGCCCAGTAAATC |
| Snail: 3’-UTR | GCCCCACAGGACTTTGATGA | CAAAAACCCACGCAGACAGG |
| Twist2: TSS | CAGCCCAGCTAGAGTTTCCAA | GAGTTGCTGGAAAGGCTCTG |
| Twist2: interior | TGAGCAAGATCCAGACGCTC | CGGAGAAGGCGTAGCTGAG |
| Twist2: 3’-UTR | GACTGGGATATGAGGACCAGC | GTAGATCTGAGTGTCCGCCC |
| β-actin: TSS | CGGCGCCCTATAAAACCCA | CTGGCCGGGCTTACCTGG |
| β-actin: interior | AAGTCCTGCCCTCATTTCCC | CACTGTGTTGGCGTACAGGT |
| β-actin: 3’-UTR | GCCGAGGACTTTGATTGCAC | TGTGTGGACTTGGGAGAGGA |
| ubiquitin C: TSS | GCTGCCACGTCAGACGAA | TACTGGGGTTCTAAGGCCGA |
| ubiquitin C: interior | AGCTGAAGCTCCGGTTTTGA | AACGGCCAGAATTTAGCGGA |
| ubiquitin C: 3’-UTR | TGCATTAGACTGGGTGAAGGG | TTGCACCCACCTTCTACAGTT |
